# Iron Dysregulation and Dormant Microbes as Causative Agents for Impaired Blood Rheology and Pathological Clotting in Alzheimer’s Type Dementia

**DOI:** 10.3389/fnins.2018.00851

**Published:** 2018-11-16

**Authors:** Lesha Pretorius, Douglas B. Kell, Etheresia Pretorius

**Affiliations:** ^1^Department of Physiological Sciences, Stellenbosch University, Stellenbosch, South Africa; ^2^School of Chemistry, The University of Manchester, Manchester, United Kingdom; ^3^The Manchester Institute of Biotechnology, The University of Manchester, Manchester, United Kingdom

**Keywords:** Alzheimer’s type dementia, iron, gut dysbiosis, iron dysregulation and dormant microbes hypothesis, inflammation

## Abstract

Alzheimer’s disease and other similar dementias are debilitating neurodegenerative disorders whose etiology and pathogenesis remain largely unknown, even after decades of research. With the anticipated increase in prevalence of Alzheimer’s type dementias among the more susceptible aging population, the need for disease-modifying treatments is urgent. While various hypotheses have been put forward over the last few decades, we suggest that Alzheimer’s type dementias are triggered by external environmental factors, co-expressing in individuals with specific genetic susceptibilities. These external stressors are defined in the Iron Dysregulation and Dormant Microbes (IDDM) hypothesis, previously put forward. This hypothesis is consistent with current literature in which serum ferritin levels of individuals diagnosed with Alzheimer’s disease are significantly higher compared those of age- and gender-matched controls. While iron dysregulation contributes to oxidative stress, it also causes microbial reactivation and virulence of the so-called dormant blood (and tissue) microbiome. Dysbiosis (changes in the microbiome) or previous infections can contribute to the dormant blood microbiome (atopobiosis^1^), and also directly promotes systemic inflammation via the amyloidogenic formation and shedding of potent inflammagens such as lipopolysaccharides. The simultaneous iron dysregulation and microbial aberrations affect the hematological system, promoting fibrin amylodiogenesis, and pathological clotting. Systemic inflammation and oxidative stress can contribute to blood brain barrier permeability and the ensuing neuro-inflammation, characteristic of Alzheimer’s type dementias. While large inter-individual variability exists, especially concerning disease pathogenesis, the IDDM hypothesis acknowledges primary causative factors which can be targeted for early diagnosis and/or for prevention of disease progression.

## Introduction

Alzheimer’s type dementia (AD) is the most prevalent example of dementia, accounting for 75% of all cases (Qui et al., 2009), with an estimated global economic impact of $604 billion per year (Langa, 2015). In 2013 this neurodegenerative disorder was diagnosed in 44.4 million individuals worldwide. This number is expected to increase to 75.6 million by 2030 (Vradenburg, 2015; Pretorius et al., 2016a). The increasing aging population and anticipated increase in prevalence of AD has severe socio-economic implications (Prince et al., 2014). Familial AD can be promoted by mutations of the amyloid precursor protein (APP), presenilin (Bu et al., 2015) and apolipoprotein E (Ayton et al., 2015) genes, however, most AD patients suffer from the sporadic form, and even after decades of research, the etiology and pathogenesis of this disease remains largely unknown (Makin, 2018), and with essentially no existing disease-modifying treatments (Vogt et al., 2017). Despite the differences between familial and sporadic AD, the resultant neuropathology is shared by both (Pritchard et al., 2017).

Vascular dementia (VaD), also known as multi-infarct dementia, is caused by obstructions in the supply of blood to the brain, which over time results in stepwise neurodegeneration (Karantzoulis and Galvin, 2011). The pathology of VaD is characterized by atheromas of primarily cerebral arteries and arterioles. Vascular lesions characteristic of VaD often co-exist with AD, with 25–80% of individuals with dementia showing both AD symptoms and cerebrovascular lesions (Jellinger, 2008), a condition termed mixed dementia (MD). In early AD, critically located small vascular lesions in subcortical regions may promote cognitive decline. This suggests a synergistic relation between disorders (Jellinger and Attems, 2007). The distinction between isolated AD, VaD, and MD, where both pathologies coexist in the same individual, remains a controversial issue and one of the most difficult diagnostic challenges (Zekry et al., 2002; Jellinger and Attems, 2007). Additionally the population-based prevalence of MD in prospective and retrospective autopsy studies, ranges from 2 to 58%, emphasizing the coexistence of AD with multiple cerebrovascular lesions in patients with cognitive impairments. Moreover inflammation and alterations of inflammatory biomarkers are common to both AD and VaD (Schmidt et al., 2002). For these reasons, VaD and the pathogenesis thereof, fall under the AD umbrella of this review.

For the individual, AD shortens life expectancy significantly, with the cognitive deficit often causing institutionalization, physical disability and reduced quality of life (Qui et al., 2009; Pritchard et al., 2017). Hallmark features of AD pathology in the brain often include extracellular plaques composed of amyloid-β (Aβ), intracellular neurofibrillary tangles which are composed of hyperphosphorylated tau, basal forebrain cholinergic insufficiency and extensive neuronal and synaptic loss in the cortex and hippocampus (Law et al., 2001; Vogt et al., 2017). Aβ42 peptides either as monomers, dimers or intermediate amyloids stimulate arrays of inflammatory gene expression characteristic of the innate immune system (Zhao et al., 2015). These Aβ42 peptides are not only highly immunogenic, but they may self-aggregate into structures that interact with the plasma membrane and cause uncontrolled seepage of ions into and/or out of the neurons (Zhao and Lukiw, 2015), disrupting their normal physiological functioning.

Another hallmark of AD is activated neuroglial cells which produce significant amounts of inflammatory molecules. At low levels this may have a protective function but the increased expression in AD could induce neurodegeneration (Kamer et al., 2008). This said, cerebral inflammation caused by the activation of glial cells is generally asymptomatic as this occurs in the normal aging brain. According to Barrientos et al. (2016) prolonged production of pro-inflammatory cytokines as well as increased neuroinflammatory responses are common in the normal aging brain. The primary source of this exaggerated response is sensitized microglia. Other causes of this phenomenon may include neuroendocrine system dysregulation and potentiation of neuroinflammatory responses following an immune challenge, due to persistently elevated glucocorticoid levels in older individuals (Barrientos et al., 2016). Excessive age-related glial priming, present in AD, could also be due to microbial infiltration from disturbed microbiomes across a permeabilized blood brain barrier, providing slow inflammatory damage (Pritchard et al., 2017).

Recent research (Dorey et al., 2014; Filiou et al., 2014; Steardo et al., 2015) also suggests that neuro-inflammation may play a central role in the pathological progression of AD. Several neuro-inflammatory mediators including reactive oxygen species, chemokines, cytokines and activated complement are produced and secreted by microglia and astrocytes in the AD brain (Pretorius et al., 2016a). While neuro-inflammation is a part of the normal aging brain, and is a hallmark of acute injury (Lucas et al., 2006) and also of infection (McColl et al., 2009), in AD excessive systemic inflammation also occurs (Pretorius et al., 2016a), simultaneously exacerbating and perpetuating neuro-inflammation. Previous studies have verified that peripheral inflammatory markers such as interferon-γ, tumor necrosis factor-alpha (TNF-α), interleukin-1β (IL-1β), and IL-6 are related to the advancement of AD (Rubio-Perez and Morillas-Ruiz, 2012; Bu et al., 2015).

**FIGURE 1:**
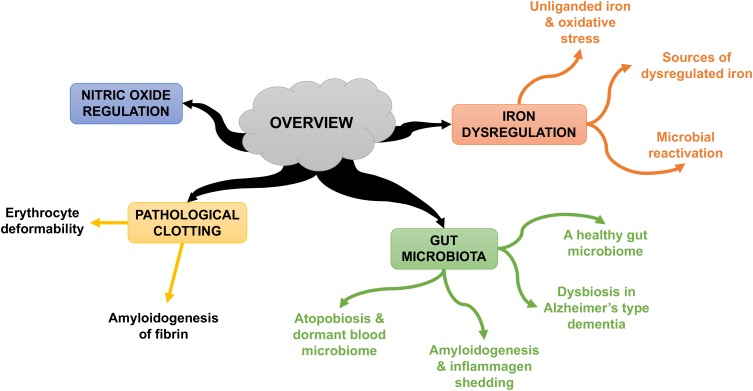
An overview of the contents of this manuscript.

Some individuals who develop AD seem to possess genetic susceptibilities that are influenced by co-occurring environmental factors (Pritchard et al., 2017). In this review, we rehearse the Iron Dysregulation and Dormant Microbes (IDDM) hypothesis, and its ability to relate events which trigger and promote the pathogenesis of inflammatory conditions such as AD. The IDDM hypothesis emphasizes two main external triggers, namely (1) stress-induced iron dysregulation, and (2) its ability to reactivate dormant or non-replicating microbes which the host had acquired via previous infections or dysbiotic communities. We believe the primary origin of such microbes comes from a dysbiotic gut (Kell and Pretorius, 2015a). These microbes are capable of sloughing off functionally significant inflammagens such as lipopolysaccharide(s) (LPS) from Gram-negative bacteria and lipoteichoic acid (LTA) from Gram-positive bacteria. The consequences of this include significant coagulopathies, for example the amyloidogenic clotting of blood, which increases cell mortality (Kell and Pretorius, 2018). The contents of this manuscript are summarized in Figure 1.

## Iron Dysregulation

Iron dysregulation is any form of deviation from normal, homeostatic iron metabolism; in particular it includes cerebrospinal fluid ferritin and serum ferritin levels as implicated in Alzheimer’s type dementias (Ayton et al., 2015). According to the Alzheimer’s Disease Neuroimaging Initiative (ADNI) cohort study, increased ferritin levels, measured in cerebrospinal fluid, were negatively correlated to cognitive performance (Mueller et al., 2005), and these levels predicted conversion of mild cognitive impairment to Alzheimer’s disease in 144 individuals over 7 years (Ayton et al., 2015). Furthermore, a strong association between ferritin levels and cerebrospinal fluid apolipoprotein E levels was observed in this study, with the major risk allele, APOE-𝜀4, inducing 22% higher levels of cerebrospinal fluid ferritin levels when compared to non-𝜀4 carriers (Ayton et al., 2015). In the same study a modest association between cerebrospinal fluid ferritin and plasma ferritin levels was noted (*p* = 0.0002). Thus, there may be great clinical relevance for the use of systemically elevated serum ferritin (SF) levels as cognitive performance markers (Kell and Pretorius, 2014; Pretorius et al., 2016a).

### Causes of Iron Dysregulation

Major sources of iron dysregulation stem from externally induced stressors (Kell and Pretorius, 2018). This form of iron dysregulation can be initiated by several factors that contribute to or cause cell death, such as mechanical damage (Zhang et al., 2013), nutritional stress (Schaffer, 2016), pharmacological stress (Primohamed et al., 2004), and of course oxidative stress (Kerley et al., 2018). Another source of free iron is via heme metabolism, due to the functioning of heme oxygenase-1 (HO-1), which catalyzes the degradation of heme (Pretorius and Kell, 2014). Since upregulation of HO-1 activity occurs in systemic inflammatory disorders in which erythrocytes are lysed, it may also be an important marker of inflammation and iron dysregulation.

**FIGURE 2:**
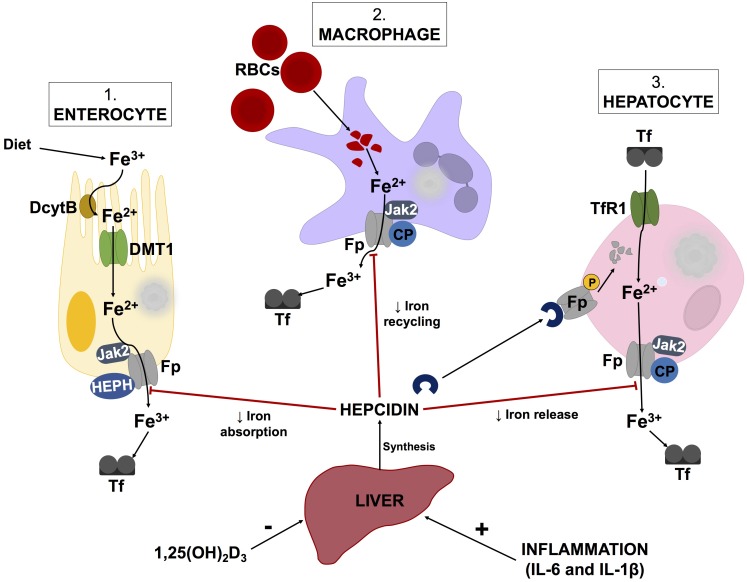
Schematic illustration of the hepcidin-ferroportin axis and its regulation of systemic iron homeostasis. Hepcidin synthesis is regulated at transcriptional level by various stimuli such as inflammatory markers and vitamin D levels. Serum ferritin concentrations are regulated by hepcidin, which causes phosphorylation, internalization and subsequent lysosomal degradation of ferroporitin (Fp), thereby reducing its expression on iron exporting cells. Adapted from Cui et al. (2009) and Mariani et al. (2009) Abbreviations: Fe^3+^, ferric iron; Fe^2+^, ferrous cation; DcytB, duodenal cytochrome B; DMT1, divalent metal transporter 1; Jak2, Janus kinase 2; HEPH, hepaestin; Tf, transferritin; RBCs, red blood cells; CP, ceruloplasmin; TfR1, transferritin receptor 1; 1,25(OH)_2_D_3_, calcitriol; IL-6, interleukin-6; IL-1β, interleukin-1beta.

Additionally, hepcidin, produced by the liver, is a key regulator of iron metabolism (Michels et al., 2015; Reichert et al., 2017). Decreases in hepcidin levels enhance surface exposure of ferroportin (Ganz and Nemeth, 2012) on enterocytes, macrophages and hepatocytes to increase serum ferritin levels (illustrated by Figure 2). Hepcidin expression is induced by inflammatory markers such as LPS, IL-1β, and IL-6, while increases in 1,25(OH)_2_D_3_ (calcitriol) levels cause hepcidin levels to decrease (Kell and Pretorius, 2018). According to a report by Bacchetta et al. (2014), decreases in hepcidin levels by 1,25(OH)_2_D_3_ are due to suppression of the *HAMP* gene by the vitamin D receptor (VDR). Chromatin immunoprecipitation assays confirmed the binding of VDR to the vitamin D response element within the proximal promotor region of the *HAMP* gene (Bacchetta et al., 2014). While this process is intricate, it appears that alterations in vitamin D metabolism could potentially instigate iron dysregulation.

Intestinal inflammation caused by gut dysbiosis can impact iron homeostasis within the GI tract (Cherayil et al., 2011), however, whether these findings have been extrapolated to serum iron homeostasis has not yet been elucidated. While iron dysregulation within the GI tract and gut dysbiosis potentially exacerbate one another, Constante et al. (2017) concluded that luminal heme originating from gastrointestinal bleeding or dietary components more likely contributes to dysbiosis of the gut microbiota in mice than *vice versa*. However, dietary non-heme iron intake from food has been associated with a 30% increased risk of Parkinson’s disease (*p* = 0.02) (Logroscino et al., 2008). In the same study authors also observed that supplemental iron intake was associated with a borderline increase in Parkinson’s disease among men (Logroscino et al., 2008). Nonetheless, the most prominent cause of iron dysregulation in the form of elevated serum ferritin levels is cell death (Kell and Pretorius, 2018).

### Unliganded Iron and Oxidative Damage

In AD, iron dysregulation and the benefits of its chelation have been known for decades (Crapper McLachlan et al., 1991), and many reviews point to the role of poorly liganded iron in AD development (Exley, 2006; Castellani et al., 2007, 2012; Kell, 2009, 2010; Sandro and Muckenthaler, 2009; Grunblatt et al., 2011; Weinreb et al., 2011; Peters et al., 2015; Wood, 2015; Belaidi and Bush, 2016). Unliganded iron and the accompanying oxidative damage is causatively related to neuro-inflammation (Bester et al., 2013; Hongkuan et al., 2015). According to Nunomura et al. (2001), neurons with neurofibrillary tangles show a 40–56% decrease in relative 8-hydroxyguanosine (8-OHG) levels when compared to neurons without neurofibrillary tangles in AD patients. 8-OHG is a marker of hydroxyl radical formation (Kell, 2009, 2010), so this finding indicates that oxidative damage is an early event in AD progression, which dissipates with lesion formation (Nunomura et al., 2001). This is further supported by (Moreira et al., 2005) who found one of the earliest pathological events in AD to be an imbalance between free radical scavenging and generation. The relationship between poorly liganded iron and oxidative stress involves iron’s catalytic contribution to the Fenton and Haber-Weiss reactions. These reactions are involved in the formation of the highly reactive hydroxyl radical (OH^-^), the most biologically detrimental free radical species, specifically with regards to macromolecules such as lipids, proteins and nucleic acids (Moreira et al., 2005; Kell, 2009, 2010; Lipinski and Pretorius, 2013; Pretorius et al., 2016a). Notably, iron chelation can reverse tau protein hyperphosphorylation in mice brains (Guo et al., 2013), thereby reducing the formation of intracellular neurofibrillary tangles.

### Microbial Reactivation

Dormancy is extremely common in microbiology, even among non-sporulating bacteria (Kaprelyants et al., 1993; Lewis, 2007; Kell et al., 2015; Kell and Kenny, 2016), for sound evolutionary reasons (Mukamolova et al., 2003). Our major hypothesis involves shared symptoms across numerous chronic inflammatory diseases caused by dormant microbes (Pretorius et al., 2016a). As reviewed in 2016 (Pretorius et al., 2016a), microbial reactivation or resuscitation, which may be autocatalytic (Mukamolova et al., 1998), indicates that these so-called dormant microbes which are non-growing and appear operationally dead (Kell et al., 1998), can recover culturability. Metals, although explicitly involved in production of free radicals and potentially oxidative stress in AD, are also involved in the process of dormant microbe reactivation (Bester et al., 2015; Kell et al., 2015). All microbes (with the possible exception of *Borrelia*) require free available iron to grow. This follows from the *in vivo* limitation of microbial growth in the absence or decreased availability of free iron (Markel et al., 2007; Reid et al., 2009; Chu et al., 2010; Nairz et al., 2010, 2014; Skaar, 2010; Haley and Skaar, 2012; Armitage and Drakesmith, 2014; Subashchandrabose and Mobley, 2015; Damron et al., 2016; Carver, 2018). It is its absence in normal metabolism that causes dormant bacteria to remain in that state. With this in mind, iron dysregulation specifically in the form of high SF levels (Kell and Pretorius, 2014), accelerates AD pathology in two ways (Pretorius et al., 2016a) which suggests the opportunity of its use as a biomarker for early diagnosis and/or a target for slowing down disease progression (Bester et al., 2013).

## Gut Microbiota

The human gut microbiome contains approximately 10–100 trillion microbes, outnumbering human genes by 100-fold (Zhu et al., 2010). The gut microbiome forms the largest diffuse organ system in the body, and is more metabolically active than is the liver (Zhao et al., 2015). Through various systemic effects, the well-being and health of the human host is largely impacted by these microbes (Zhao and Lukiw, 2015). However, the human microbiome displays a high degree of variation at inter-individual levels (Costello et al., 2009). For example, even as infants the composition of the gut microbiota is idiosyncratic with significant inter-individual variation being evident from the 1st day after birth (Chong et al., 2018). As a consequence it is has been impossible to define a healthy microbiome (Dave et al., 2012), but, it has become apparent that certain compositions of microbial communities may be able to promote both health and disease (Blum, 2017).

Intriguingly the human gastrointestinal (GI) tract has co-evolved with two major phyla: (1) *Firmicutes* which constitutes 80% and, (2) *Bacteriodetes* which constitutes 20% of all GI tract bacteria (Pritchard et al., 2017; Zhao et al., 2017). There is increasing evidence implicating host–microbiome interactions at all stages of complexity including the central nervous system (Stilling et al., 2014). According to Stilling et al. (2014) gut-microbial products can distress chromatin plasticity, leading to changes in neuronal transcription and host conduct. This is due to the fact that the microbiota is an essential mediator of gene–environment interactions and may themselves be regarded as an epigenetic entity. Current research which supports this view includes reports which have characterized small non-coding RNA secreted from microbial cells in the GI tract (Ghosal et al., 2015; Zhao et al., 2017).

Alterations of the gut microbiota can promote pro-inflammatory cytokine release and increase intestinal permeability, which has also been associated with AD (Pistollato et al., 2016). In particular, changes of gut microbial diversity and density can result in systemic and neuro-inflammation as well as dysfunction of the cerebellum and hippocampus (Pistollato et al., 2016). Considering the multitudes of LPSs and amyloids in the GI tract, it is plausible that the microbiota is involved in the pathogenesis of neurological disorders hallmarked by amyloidogenic features (Tran and Greenwood-Van Meerveld, 2013; Shoemark and Allen, 2015; Zhao and Lukiw, 2015) such as AD.

### Dysbiosis

Dysbiosis refers to alteration in the composition of the microbiota and has been implicated in the etiology of various disease states (de Oliveira et al., 2017; Kho and Lal, 2018). Perturbations of the gut microbiota can occur as consequences of antibiotic treatment (especially during infancy), dietary changes, sedentary behavior, food additives, non-steroidal anti-inflammatory and other drugs (Wallace, 2013; Ticinesi et al., 2017; Maier et al., 2018) and various other conditions (Pistollato et al., 2016). Dietary changes, for example, may explain up to 57% of the total structural variation the gut microbiome (Brown et al., 2012). Using a humanized mouse model in which adult human fecal microbiota were transplanted into germ-free mice, Brown et al. (2012) reported a shift in the composition of microbiota after switching the mice from a low-fat, plant polysaccharide-rich diet to a Western diet. While diets rich in refined sugars result in different changes to the microbiota from diets lacking fiber, due to the intricate balance that exists between microbial species, alterations in one species can disrupt the entire microbial community (Brown et al., 2012). Thus, alterations in diet, although modifiable, could potentially be a major hurdle in treatment dysbiosis-related conditions if not adequately addressed. Furthermore, physical activity and its relative intensity can induce or prevent gut dysbiosis by influencing splanchnic blood flow, intestinal permeability and inflammatory cytokine expression (Monda et al., 2017). For example, in mice, even in combination with a high-fat diet, exercise may reduce inflammatory infiltrate into epithelial cells, protecting the integrity of the GI wall (Campbell et al., 2016).

Broad-scale fluctuations in the gut microbiota play significant roles in disease advancement through immune and systemic activation (Vogt et al., 2017). There is considerable evidence for the existence of the gut-brain axis which allows bi-directional communication through various pathways such as neural, endocrine and immune mechanisms (Gareau, 2014; Lyte, 2014; Bienenstock et al., 2015; Köhler et al., 2016; Fung et al., 2017; Sandhu et al., 2017). Within this context, changes in microbial communities could lead to pathophysiological changes in the brain of individuals with AD. Dysbiosis is often associated with increased anxiety and memory impairment due to decreased secretion of neurotrophic factors such as brain-derived neurotrophic factor (Stilling et al., 2014).

#### Dysbiosis in Alzheimer’s Type Dementia

The recognition of dysbiosis and its possible link to neurodegenerative diseases are increasing as our understanding about the gut-brain axis improves (Miklossy and McGeer, 2016). A recent study in which transgenic AD mice were raised under germ-free conditions, indicated that these mice, compared to conventionally-raised AD mice, had less cerebral amyloid deposition (Harach et al., 2017). This indicates that a dysbiotic gut may influence progression of amyloid pathologies.

The gut microbiome diversity of AD patients is notably decreased and compositionally distinct from age- and gender-matched controls (Vogt et al., 2017). In this study Vogt et al. (2017), the authors reported decreases in Firmicutes and *Bifidobacterium* spp. and increased Bacteriodetes in AD patients. *Bifidobacterium* spp. are important in gut health and their beneficial effects are well-documented (Arboleya et al., 2016). For instance, particular *Bifidobacterium* species, such as *B. bifidum, B. cantenulatum, B. breve*, and *B. adolescentis*, have anti-inflammatory properties such as inhibiting LPS-induced IL-8 production and TNFα expression (Khokhlova et al., 2012) Bifidobacteria are also known to reduce intestinal permeability (Underwood et al., 2015). Moreover, *Bifidobacterium* supplementation has shown to decrease intestinal LPS levels and mend the GI-mucosal barrier in mice (Griffiths et al., 2004; Wang et al., 2006). The phylum Bacteriodetes encompasses an abundant and diverse group of Gram-negative bacteria, which have been detected as being increased in patients with Parkinson’s disease (Keshavarzian et al., 2015). The shedding of LPS and subsequent induction of inflammation is also associated with the increase in numbers of fraction of these bacteria. Considering these findings, individuals with AD may present with a gut microbial phenotype that has an increased propensity for the translocation of inflammatory bacterial components (Vogt et al., 2017).

### Amyloidogenesis

Amyloid by definition is protein that is deposited as an insoluble fibril as a result of successive alterations in the protein folding process, known as amyloidosis. Similarly to prions, there is no change in the primary amino acid sequence of the proteins when they adopt an insoluble amyloid form (Kell and Pretorius, 2018). Interestingly, self-associating amyloidogenic lipoproteins are secreted by most microbial species (Schwartz et al., 2016), and amyloid is one of the main secretory products of the gut microbiome (Zhao et al., 2017). The role of the gut in dissemination of amyloid proteins has been reviewed by Pistollato et al. (2016), in which the authors adopted the unifying prion concept to explain transmission of these prion-like proteins. The cumulative life-long contribution of microbial amyloid to neurodegenerative pathophysiology is little recognized. Progressive production and aggregation of amyloids contributes to the pathogenesis of diseases in which amyloids accumulate, which is often mediated by microglial cells (Zhao et al., 2017). Thus, bacterial amyloids originating from the gut microbiome may enhance inflammatory responses to cerebral accumulation of Aβ and play a role in the pathogenesis of AD (Pistollato et al., 2016).

For example, functional amyloid from *Klebsiella pneumoniae* can depolymerise from its fibrillary state to release oligomers which induce cytotoxicity equal to that caused by pathological Aβ in AD (Shahnawaz and Soto, 2012). The molecular mimicry theory (Ebringer and Rashid, 2009; Proal et al., 2013, 2017) includes microbial curli fibers^2^ and Aβ as a protein–protein interaction which could result in cross-seeding, even if these proteins are essentially unalike (Friedland, 2015; Zhao et al., 2015). These extrinsic curli proteins act as pathogen-associated molecular patterns with increased β-pleated sheet structures, and can cross-react with antibodies to Aβ plaques (Miklossy, 2016). It is plausible that extracellular senile plaques, due to their morphological appearance and density, may in fact be constituted of curli-like Aβ compositions. In theory, this gives bacterial phylotypes a more blatant role in AD causality (Pritchard et al., 2017). The influence of gut microbiota on amyloid formation and propagation is more significant throughout aging as both the epithelium of the GI tract and the blood-brain barrier (BBB) increase in permeability to smaller molecules (Tran and Greenwood-Van Meerveld, 2013; Pistollato et al., 2016).

### Shedding of Inflammagens

Some of the foremost bacterial species of the GI tract, such as the Gram-negative bacilli *Bacteroides fragilis* and *Escherichia coli*, secrete a multifarious selection of pro-inflammatory neurotoxins which are not only pathogenic but also detrimental to the homeostatic functioning of neurons (Zhao et al., 2017). The shedding of inflammagens such as LPS from gut-microbiota-related dysbiosis causes systemic and neuro-inflammation and promotes gut permeability. Shedding can occur in response to varying physiological and environmental signals with the most extreme example of microbial shedding known as the Jarisch-Herxheimer reaction. This is basically an uninhibited cytokine storm, which is caused by prompt release of inflammagenic material, often following antibiotic treatment of syphilis (Kell and Pretorius, 2018), or other diseases caused by spirochetes. As mentioned earlier, this may promote neuronal dysfunction and apoptosis, impairment of synaptic plasticity and induced vulnerability to cognitive decline (Daulatzai, 2014). Bacterial LPS has also been found in AD hippocampal brain lysates where the mean LPS levels were three-fold higher in the hippocampus of AD patients than in age-matched controls. This was said to increase to 26-fold in advanced AD hippocampal cases (Zhao et al., 2017).

LPSs are sparingly soluble but over a period of time characteristically form large heterogenous aggregates which are exceptionally immunogenic (Zhao et al., 2015). Interestingly the glycosylphosphatidyl-inositol-anchored LPS and microbe-detecting CD41 receptor, which is crucial in the neutralization of invading microbes, is similarly, stimulated by Aβ fibrils (Dowhan, 2014), relating innate-immune signaling with amyloidogenesis in AD. It has also been noted that previous bacterial infections which resulted in the formation of antibodies to amyloids or bacterial inflammagens may predispose central nervous system amyloids to ensuing attack by antibodies, which results in upregulation of neuro-inflammation (Via et al., 2015; Zhao et al., 2015). The inflammagenic potency of LPS is so pronounced that it is frequently used to stimulate *in vivo* AD models (Lee et al., 2008; Catorce and Gevorkian, 2016; Zakaria et al., 2017).

LTA is the cell wall equivalent of LPS in Gram-positive bacteria and is equally capable of inducing an inflammatory response through its interactions with toll-like receptor (TLR) 2 (Kell and Pretorius, 2018). LTA has not been as well studied as LPS with regards to inflammagenesis, however, recent work suggests that LTAs may in fact be more potent than LPSs (Pretorius et al., 2018). The stimulation of inflammatory cytokine production by both LPS and LTA are mediated through TLR binding (reviewed by Kell and Pretorius, 2018). In response to inflammagens, concentrations of acute phase biomarkers and inflammatory cytokines such as TNF-α, IL-1β, IL-6, IL-8, C-reactive protein (CRP), serum amyloid A (SAA), and fibrinogen increase significantly (deRosset and Strutz, 2015; De Buck et al., 2016; Pindjakova et al., 2017).

Additionally, increased BBB permeability typically observed in individuals with AD can be caused by shed bacterial inflammagens. The BBB plays a fundamental role in the initiation and continuance of chronic inflammation during AD (Zenaro et al., 2017). While Aβ accumulation in vasculature results in inflammatory events that increase BBB permeability in AD (Erickson and Banks, 2013), proteolytic enzymes such as carbonic anhydrases, peptidyl deiminases and gingipains, and appendages such as curli fibers, fimbriae and other amyloid-like proteins, which are carried with LPS (Pritchard et al., 2017) and contribute significantly to BBB permeabilization. BBB dysfunction during AD effects Aβ clearance, endothelial cell function, tight junction integrity and may activate glial cells which accelerates migration of leukocytes to the brain (Zenaro et al., 2017). This could stimulate the beginning of chronic neuro-inflammation in AD.

### Atopobiosis

**FIGURE 3:**
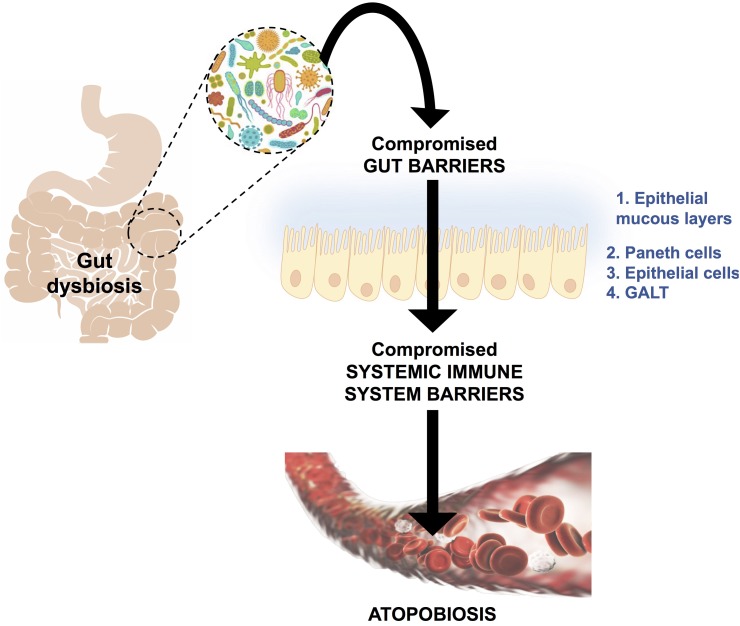
A schematic representation of bacterial translocation from a dysbiotic gastrointestinal tract to normally sterile tissues, such as blood. This occurs due to compromised gut barriers such as the epithelial mucous layer, Paneth cells (which secreted antimicrobial peptides) and epithelial cells and gut associated lymphoid tissue (GALT) as mechanical barriers. Simultaneously, there will be compromised systemic immune system function, which enables microbes to enter into normally sterile tissues via dendritic cells, injured epithelial, M-cells and Peyer’s patches. This phenomenon is known as atopobiosis and is implicated in both communicable and non-communicable diseases. Figure adapted from Potgieter et al. (2015).

Atopobiosis refers to the appearance of some of the gut microbes in the wrong place (Potgieter et al., 2015). The sterility of blood is seemingly a controversial topic. The presence of a “physiological” blood microbiota has been reported by several studies using 16S ribosomal DNA quantitative polymerase chain reactions (Amar et al., 2013; Lelouvier et al., 2016; Martel et al., 2017; Proal and Marshall, 2018). Yet, in these studies the plausible source of these microbes from gut permeability was not investigated. Authors of the same studies have reported that changes in the composition of this blood microbiota can be associated with disease. However, based on immunological and clinical understanding (Ochei and Kolhatkar, 2000) the detection of bacteria in blood is always abnormal (Kinnby and Chavez de Paz, 2016; Andreadou et al., 2017) and thus referred to as atopobiosis (Potgieter et al., 2015). As seen in Figure 3, atopobiosis is caused by bacterial translocation in which bacteria move from the GI tract to normally sterile blood and tissues (Potgieter et al., 2015). Once translocation has taken place, these bacteria can cause direct infection and inflammation. The process of bacterial translocation has been reviewed by Potgieter et al. (2015) but can briefly be described by the simultaneous dysfunction of three role players: (1) dendritic cells (2) GI epithelium and, (3) M cells which overlay Peyer’s patches. It is a combination of various compromised mucosal defenses that leads to bacterial translocation. When the influx of gut microbes and toxins is particularly abundant, it is often referred to as a “leaky gut” (Maes, 2009; Fasano, 2012; Quigley, 2016; Mu et al., 2017; Kell and Pretorius, 2018) and is due to severely compromised epithelial tight junctions (Fasano, 2012).

### Dormant Blood Microbiome

**FIGURE 4:**
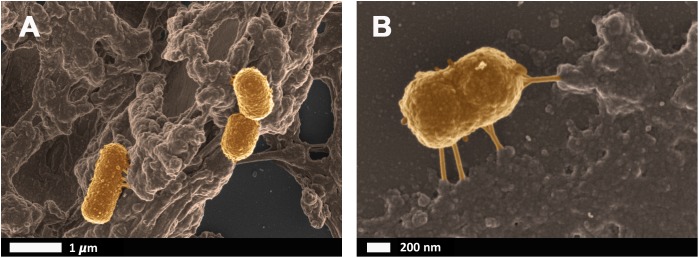
Scanning electron micrographs of bacteria associated with pathological coagulations found within the blood of individuals diagnosed with Alzheimer’s disease. Unpublished data from Potgieter et al. (2015). **(A)** low magnification, **(B)** higher magnification.

Bacteria that have successfully translocated to the blood have the ability to become dormant and reactivate upon appropriate stimuli, such as iron dysregulation in the form of high SF levels as discussed in section Causes of Iron Dysregulation. In contrast to conventional infections, these bacteria do not multiply but enter dormant states (Kell and Kenny, 2016; Pretorius et al., 2016a; Kell and Pretorius, 2018). This is especially relevant in clinical settings when toxic concentrations of antibiotics promote bacterial persistence or adoption of a dormant state that permits their survival (Kell and Pretorius, 2018). Even though the major source of this dormant blood microbiome has been reviewed here as a dysbiotic and permeabilized GI tract, we also recognize periodontitis, gingivitis, urinary tract infections and even insemination as possible points of microbial infection and subsequent translocation (Kamer et al., 2008; Flores-Mireles et al., 2015; Kenny and Kell, 2017; Pretorius et al., 2017; Pritchard et al., 2017). Current research has drifted toward the correlation between an individual’s infectious burden and risk of developing AD, with particular emphasis on herpes simplex virus 1 (HSV1) and human herpesvirus 6 and 7 infections (Honjo et al., 2009; Itzhaki, 2014, 2017; Bu et al., 2015; Itzhaki et al., 2016; Itzhaki and Tabet, 2017; Eimer et al., 2018; Itzhaki and Lathe, 2018; Readhead et al., 2018; Tzeng et al., 2018). Dormant bacteria can survive in leukocytes (Thwaites and Gant, 2011; Liehl et al., 2015) and erythrocytes (Potgieter et al., 2015) to establish the dormant blood microbiome. In Potgieter et al. (2015) transmission electron micrographs of cellular inclusions thought to be L-forms of bacteria inside erythrocytes of individuals diagnosed with AD can be seen (see also Mattman, 2001). Figure 4 illustrates the association of bacteria with pathological coagulations within the blood of individuals diagnosed with AD. This dormant blood microbiome affects the integrity of the hematological system, promoting pathological coagulation.

## Pathological Coagulation

LPS is well known to cause inflammatory cytokine production which can cause hypercoagulation, often referred to as endotoxin-mediated hypercoagulation (Slotta et al., 2008; Kell and Pretorius, 2017). LPS may activate the coagulation pathway via upregulation of tissue factor (TF), which leads to activation of pro-thrombin (Landsem et al., 2015). Two main components of the hematological system namely fibrinogen and erythrocytes and the changes induced during inflammation and AD, will be discussed below.

### Fibrinogen

**FIGURE 5:**
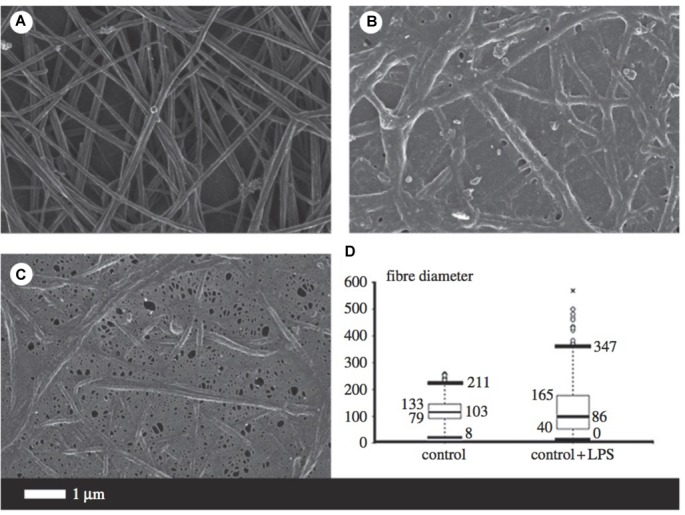
Micrographs from scanning electron microscopy **(A–C)** as well as a box and whisker plot indicating fiber distribution **(D)** taken from Pretorius et al. (2016b) (open access publication). **(A)** Is indicative of healthy fibrin fibers during coagulation (with the addition of thrombin), while **(B,C)** demonstrate the large effect of 0.2 ng/l^-1^ 0111:B4 LPS on fibrin morphology. LPS, lipopolysaccharide. ∗, outlier measurement.

Fibrinogen is the precursor of fibrin and thus plays a major role in thrombosis and hemostasis (Pretorius and Kell, 2014). During inflammation, circulating fibrinogen levels are increased, while fibrinolysis is impaired. Amyloidogenesis of the fibrin fibers occurs, resulting in the formation of dense matted deposits which trap other blood cells and distort their shape. The combination of the increased propensity to form a stiffer fibrin network and reduced fibrinolysis is a feature of numerous inflammatory diseases such as AD (Ahn et al., 2010; Zamolodchikov and Strickland, 2012). Figure 5 illustrates some of the morphological changes of fibrin fibers due to the addition of LPS.

The presence of excessive (unliganded) iron and the consequential production of hydroxyl radicals is one of the leading causes of altered fibrin cross-linking (Pretorius and Kell, 2014; Kell and Pretorius, 2015b). Dense matted deposits produced in the presence of ferric ions are abnormally resistant to chemical and proteolytic degradation, hypothesized to be due to the existence of intermolecular hydrophobic bonds (Lipinski and Pretorius, 2012). As reviewed, it seems that iron dysregulation may serve as a causative factor for multiple pathologies involved in AD progression. This altered fibrinogen structure, with excessive cross-linking and increased β-pleated sheet structure, has been implicated in the development of neuro-inflammation and memory impairments. Increased fibrinogen levels are a strong indicator of cerebrovascular risk since fibrinogen binds to Aβ which further delays clot degradation (Bester et al., 2013; Pretorius et al., 2016a). In addition, pharmacological inhibition of the fibrinogen–Aβ interaction with Ru-505 altered thrombus structure and paused cognitive decline in mice (Ahn et al., 2014), providing a strong association between pathological clotting and neurodegenerative diseases.

### Erythrocytes

The dynamics of erythrocytes represent an important but understudied feature of the cardiovascular system. Central to this is their rheology, viscosity, aggregation and deformability (Baskurt et al., 2011; Pretorius and Kell, 2014). It is thought that alterations in erythrocytes and their rheology can contribute to the pathogenesis of AD by hindering oxygen delivery (Tripathy et al., 2013). Dense matted deposits may also trap erythrocytes, impairing their effective delivery of oxygen to the brain. Proficient oxygen delivery is vital for normal functioning of the brain, underpinned by that fact that 20% of an individual’s total oxygen intake is used by the brain. Neurons are particularly susceptible to hypoxic periods, which can cause irreversible neurological consequences (Lipinski and Pretorius, 2013) after only a few minutes. Altered functioning of erythrocytes is also strongly suggested by changes in their size distribution (Ozturk et al., 2013; Weuve et al., 2014; Pilling et al., 2017; Winchester et al., 2018).

**FIGURE 6:**
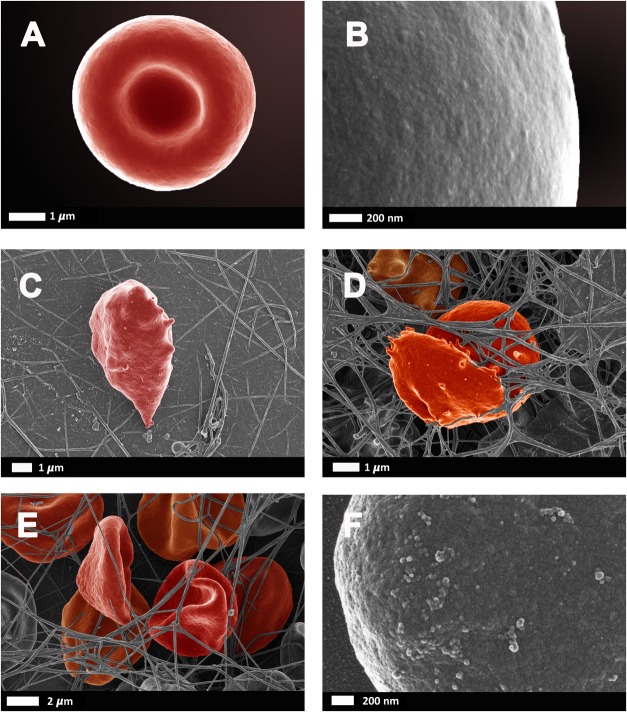
Erythrocytes from healthy individuals **(A–B)** and individuals diagnosed with Alzheimer’s type dementia **(C–F)**. **(C)** Serum ferritin: 57 ng.L^-1^ **(D)** Serum ferritin: 256 ng.L^-1^ **(E)** Serum ferritin: 302 ng.L^-1^ **(F)** Serum ferritin: 302 ng.L^-1^ (100,000× machine magnification of erythrocyte membrane). Unpublished data from Bester et al. (2013).

As reviewed by Pretorius and Kell (2014), erythrocyte deformability is a complex process which is significantly altered by pathophysiological conditions. Briefly, reduced erythrocyte deformability is an important feature of inflammation in which reactive oxygen species cause degradation of spectrin and band 3, for example, which are important membrane proteins. Since the plasma membrane and the cytoskeleton are responsible for the preservation of erythrocyte shape and stability, modifications of the phospholipid bilayer can affect deformability. In 2010, Mohanty et al. (2010) reported that 15% of erythrocytes in AD were elongated and suggested that a potential link between changes in the erythrocyte proteome could contribute to AD pathology. Deformability and impaired reformability, particularly after erythrocytes have moved through capillaries, reduces their oxygen carrying capacity, potentiating transient states of hypoxia. Figure 6 illustrates erythrocyte deformability as well as loss of membrane integrity in typical blood samples from individual’s diagnosed with AD. It is clear to see how the loss of the typical bi-concave structure of these erythrocytes would impair their physiological functioning.

## Nitric Oxide Regulation

The dysregulation of nitric oxide (NO) synthesis is prevalent in many diseased conditions (Cooke and Dzau, 1997; Kolios et al., 2004; Naseem, 2005; Karpuzoglu and Ahmed, 2006; Pacher et al., 2007). Recently, the physiological role of erythrocytes in regulating nitric oxide levels has also been investigated (Saldanha, 2016). Due to the consequences of chronic systemic inflammation and pathological coagulation, such as erythrocyte deformability and lysis, it is plausible that aberrant NO regulation could occur and causatively contribute to AD pathogenesis.

NO is synthesized by three isoforms of nitric oxide synthases. Although NO has vasoactive, immunological and neurophysiological functions, it can be neurotoxic at high concentrations and has been implicated in neurodegenerative diseases (Law et al., 2001). In this regard, erythrocytes have an important role in vascular function. Normally, erythrocytes regulate hemostasis by balancing oxygen delivery and NO scavenging and production. More specifically, hemoglobin (Hb) compartmentalization and encapsulation reduces an erythrocyte’s ability to scavenge NO. However, in various diseases hemolysis introduces cell-free Hb into circulation, increasing NO scavenging and inducing hypertension (Helms et al., 2018). A decrease in NO may lead to a reduced ability to learn and memorize due to impairment of long-term potentiation, since NO is responsible for the synaptic efficiency of pre-synaptic glutamatergic neurons (Panthi et al., 2018). Hypoperfusion of the brain and increased oxidative stress within the vasculature are common phenomena in AD (Edwards and Rickard, 2007) and some researchers have reported abnormalities in NO synthase expression as early symptoms of cognitive impairment and AD (Lüth et al., 2002; Malinski, 2007).

Furthermore, NO can exert its neurotoxic effects through three main mechanisms. Firstly, NO is a free radical which can react with superoxide anions to produce another damaging radical, peroxynitrite (Eliasson et al., 1999). This can produce significant oxidative stress which leads to oxidative damage and eventually neuronal death. Secondly, NO causes nitrosylation in a variety of proteins such as glyceraldehyde-3 phosphate dehydrogenase (GADPH) and protein kinase C (PKC), inhibiting their functioning. Lastly, NO can impair glycolysis and the latter energy manufacturing by potentiating ADP-ribosylation of GADPH (Law et al., 2001) as well as binding aconitase and thereby inhibiting electron transfer in the electron transport chain.

Together, pathological coagulation and altered nitric oxide regulation can enhance the propensity of spontaneous clot formation in cerebral arteries and arterioles. These clots are more resistant to degradation, due to amylodiogenesis of fibrin fibers in the presence of ferric irons, and may thus potentiate transient periods of hypoxia in the brain, promoting the occurrence of MD.

## Conclusion

**FIGURE 7:**
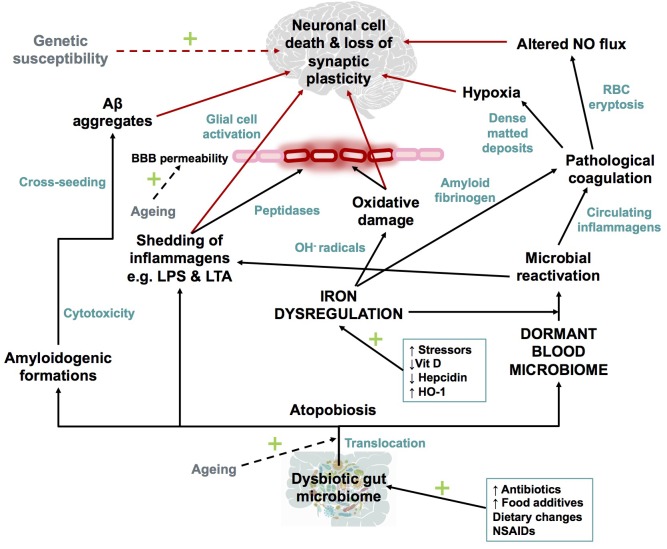
Schematic representation of The Iron Dysregulation and Dormant Microbes hypothesis. With reference to the scheme it is plausible that systemic inflammation caused by stress induced iron dysregulation and subsequent microbial reactivation can lead to increased permeability of the blood brain barrier as well as a neuro-inflammatory environment which promotes neuronal cell death. The red arrows indicate factors which could contribute to synaptic loss and neuronal death. Dashed arrows have not been discussed in detail in this review but are recognized as contributors to the pathogenesis of Alzheimer’s-type dementias. Adapted from Kell and Pretorius (2018). Abbreviations: Aβ, amyloid beta; NO, nitric oxide; BBB, blood brain barrier; RBC, red blood cell; OH^-^, hydroxyl; LPS, lipopolysaccharide; LTA, lipoteichoic acid; HO-1, heme oxygenase-1 and NSAIDs, non-steroidal anti-inflammatory drugs.

While numerous hypotheses for the pathogenesis of AD exist (extensively reviewed by Law et al., 2001; Hardy and Selkoe, 2002; Swerdlow, 2007; Armstrong, 2011;

Dong et al., 2012; Barage and Sonawane, 2015; Selkoe and Hardy, 2016; Area-Gomez and Schon, 2017; Makin, 2018), the IDDM hypothesis (illustrated in Figure 7) recognizes that a combination of a dormant blood or tissue microbiome can be re-activated by unliganded iron. This in turn can induce shedding of highly inflammagenic LPS and LTA molecules, from which all known sequlae of AD can be seen to follow.

As reviewed here, dysbiotic bacterial communities from the GI tract or microbes from other sources of infection, translocate into normally sterile tissues and blood to establish a dormant blood microbiome. Characteristic of AD (and many other chronic, inflammatory diseases; Kell, 2009) is iron dysregulation, that amongst many other affects, causes microbial reactivation. Iron dysregulation, specifically in the form of elevated SF levels, which can causatively modulate oxidative damage, the formation of amyloidogenic fibrinogen and the aforementioned microbial reactivation as part of AD pathogenesis. Microbes contribute to amyloidogenic formations, inflammagen shedding, pathological clotting and systemic as well as neuro-inflammation. Various changes to the hematological system ensue, increasing the risk of pathological coagulation and transient hypoxic events in the AD brain. Increasing BBB permeability and glial cell activation initiate the slow inflammatory progression of AD, in combination with loss of synaptic plasticity and neuronal death. While AD is a disease too complex to fit to any particular model, the IDDM hypothesis highlights several causative role players which can be targeted for early diagnosis and/or for prevention of disease progression.

## Consent for Publication

All authors have read the paper and agree that it can be published.

## Author Contributions

LP wrote the paper. DK co-wrote the paper and edited the paper. EP co-wrote the paper and is the study leader.

## Conflict of Interest Statement

The authors declare that the research was conducted in the absence of any commercial or financial relationships that could be construed as a potential conflict of interest.

## Footnotes

2 Extracellular bacterial amyloids.
